# Alkaline Hydrolysis and Dyeing Characteristics of Sea-Island-Type Ultramicrofibers of PET Tricot Fabrics with Black Disperse Dye

**DOI:** 10.3390/polym12061243

**Published:** 2020-05-29

**Authors:** Jeong Min Kang, Min Gu Kim, Ji Eun Lee, Jae Wang Ko, Il Jin Kim, Jae Yeon Lee, Dong Jin Lee, Seong Ik Ko, Dae Ho Jung, Seung Geol Lee

**Affiliations:** 1 Department of Organic Material Science and Engineering, Pusan National University, Busan 46241, Korea; kdkd7892@pusan.ac.kr (J.M.K.); kimmingu95@pusan.ac.kr (M.G.K.); ahdwkdrn@pusan.ac.kr (J.E.L.); 2 Korea Institute of Footwear and Leather Technology, Busan 47154, Korea; jwko@kiflt.re.kr (J.W.K.); ijkim@kiflt.re.kr (I.J.K.); jylee@kiflt.re.kr (J.Y.L.); dongjlee@kiflt.re.kr (D.J.L.); 3 R&D Institutes, Jeongsan International Co., Ltd., Busan 46756, Korea; siko@ep.jeongsan.com (S.I.K.); dhjung@ep.jeongsan.com (D.H.J.)

**Keywords:** ultramicrofiber, PET, dyeing, alkali hydrolysis, disperse dye, black color

## Abstract

In this study, we investigated conditions for the alkaline hydrolysis and black-disperse dyeing of sea-island-type polyethylene terephthalate (PET) ultramicrofiber tricot fabric. We examined the weight loss ratios and tensile strengths according to the NaOH content (10–30% on mass of fabric (omf)) during treatment; the optimal conditions used 25% omf NaOH for 30 min at 100 °C for an average weight loss ratio of 23.47%. By scanning electron microscope (SEM) analysis, the ‘sea’ components are extracted with increasing NaOH concentration until 25% omf NaOH, and damage of the ‘island’ components above 25% omf NaOH leads to a reduction in tensile strength. The dyeing conditions, including temperature (95–135 °C), time (20–60 min), pH buffer solution concentration (1–9 g/L), and contents of dispersant (1–9 g/L) and UV-absorbent (5–25% omf) were also explored. The optimal dyeing conditions were established as a dye concentration of 8% omf with 1 g/L dispersant, 1 g/L pH buffer solution concentration, and 10% omf UV-absorbent at 135 °C for 40 min at a 1:10 goods-to-liquor ratio. The rubbing colorfastness values for the fabrics dyed with the black disperse dye spanned four grades under dry and wet conditions. The light colorfastness values of the dyed fabrics were good to excellent in the range of 4–5 grades.

## 1. Introduction

The increasing demand for high-quality textile materials, especially in the automobile and leisure industries, has driven the development of high-value-added synthetic fibers in which the advantages of synthetic and natural fibers are supplemented [1,2,3,4]. For example, the consumption of modified polyethylene terephthalate (PET) fibers, which are economical and exhibit excellent mechanical properties and chemical stability, has grown substantially in recent years [4,5,6,7,8,9]. To address the problem of uniformly textured PET fibers, the development of properties such as soft texture, flexibility, glossy drape, and absorptivity similar to those of nylon and rayon has been an engaging subject of study [10]. The modification of PET fibers is essential to improve their dyeability with water-soluble dyes by increasing the hydrophilicity of the fiber surface under strongly alkaline conditions [1,2,11,12,13,14].

Microfibers are usually defined as having filaments of 0.04–0.4 denier [15,16,17]. Generally, two methods are used to produce microfibers, direct spinning or conjugate spinning. In direct spinning, a single component filament is extruded through a spinneret, whereas in conjugate spinning, filaments comprising of two or more components are spun and then separated by dissolution after spinning. Therefore, the fibers that are extruded by conjugate spinning ultimately have a finer denier than those by direct spinning. The finest microfibers produced by the dissolution technique are known as sea-island-type microfibers; the cross-sections of these yarns resemble islands (the regular polyester) floating in the sea (the alkali-soluble polyester). After the ‘sea’ component is dissolved away with alkaline solvent, the ‘island’ polymer that remains forms the finest fibers. If the alkaline hydrolysis is insufficient, the ‘sea’ component is not completely dissolved and microfibers are not obtained [18,19,20]. When the alkaline hydrolysis is excessive, all of the components can be damaged, resulting in lower tensile strength [21]. Therefore, the optimal alkaline hydrolysis conditions for removing the ‘sea’ component are important for producing quality fabrics.

In this study, we investigated the optimal alkali-treatment conditions for sea-island ultramicrofibers of PET tricot fabric. The optimal conditions for the hydrolyzed PET fabric are also key factors influencing the final product, because the alkali-treated ultramicrofibers consume larger amounts of dye during colorizing due to their larger surface area per unit weight [22,23]. This leads to decreased *K*/*S* values (ratios of the absorption and scattering coefficients (*K* and *S*, respectively)) and lower light, rubbing, and washing colorfastness properties with increased dye contents. Furthermore, with respect to colorfastness, it is difficult to achieve a high degree of washing colorfastness for deep colors such as black in PET fabrics because of their high refractive index and high surface reflectivity [14]. To obtain the optimal dyeing conditions for black coloration in PET fabrics, we investigated the dyeing process of sea-island-type ultramicrofiber PET fabric as a function of the following conditions: dyeing temperature (95–135 °C), dyeing time (20–60 min), dye concentration (2–10% on mass of fabric (omf)), pH buffer solution concentration (1–9 g/L), and the contents of dispersant (1–9 g/L) and UV absorbent (5–25% omf). We also examined the *K*/*S* values and CIE L^*^a^*^b^*^ coordinates of the dyed PET fabrics to evaluate the black color. Finally, the rubbing and light colorfastness properties of the dyed PET fabrics were evaluated.

## 2. Experimental

### 2.1. Materials

We used the sea-island ultramicrofibers of a PET tricot fabric knitted using a 2-bar tricot knitting machine and knife-treated after immersion in silicone resin (PET, 50 denier/36 filaments; co-PET, 75 denier/24 filaments; sea-island ratio PET:co-PET = 7:3; 36 islands, 0.06 denier per island, provided by Jeongsan International Co. Ltd., Busan, Korea). The fabric was dyed with anthraquinone-based disperse Dianix Black AM-SLR dye (Dystar Korea Ltd., Ansan, Korea), which exhibits high light-colorfastness properties as a black disperse dye and is mainly used commercially without purification. Appropriate amounts of other chemical reagents—polyoxyethylene alkyl ether sulfate (ECOVINN DA-DLP, ICEI Woobang Co., Yangsan, Korea) as a dispersant, citric acid (ECOVINN AB, ICEI Woobang Co.) as a pH buffer solution, and a UV-absorbent (BENESOL UVA-EPM, ICEI Woobang Co.)—were used for the alkaline hydrolysis and dyeing processes.

### 2.2. Alkaline Hydrolysis

**Figure 1:**
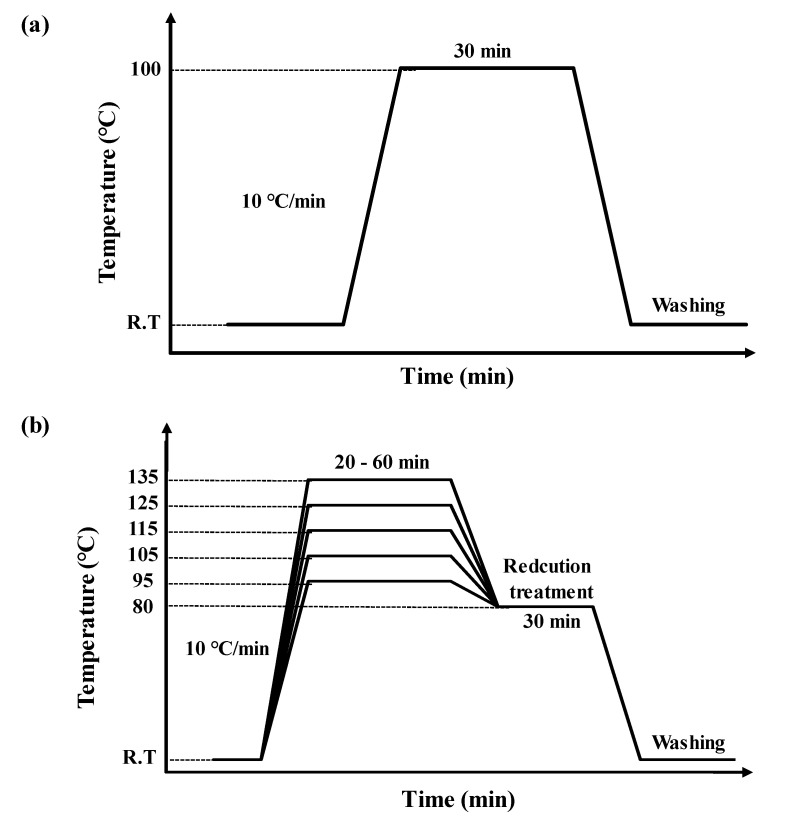
(**a**) Alkaline hydrolysis profile for various concentrations of NaOH. (**b**) Dyeing profiles of sea-island ultramicrofibers of PET tricot fabrics.

To confirm the optimum alkaline conditions, we examined the weight reduction process with different NaOH concentrations (10–30% omf) at a 1:10 goods-to-liquor ratio using an IR dyeing machine (DL-6000, Daelim Starlet Co., Siheung, Korea) with increasing temperature (10 °C/min). The alkali-treatment time was 30 min. After treatment, the fabrics were neutralized with aqueous acetic acid solution (2 g/L, Junsei Chemical Co. Ltd., Tokyo, Japan), rinsed with distilled water, and then dried at 130 °C for 5 min (Figure 1a) in an oven (CO-72, Dongwon, Busan, Korea). The front and back surfaces of the alkali-treated fibers were observed using a polarizing microscope (Eclipse LV100 POL, Nikon, Tokyo, Japan). The weight loss ratios were calculated using Equation (1):

(1) $$\mathrm{Weight}\;\mathrm{loss}\;\left(\%\right)=\frac{\left(W_{0}-W_{R}\right)}{W_{0}}\times 100$$

where *W*_0_ denotes the weight of fabric before alkali treatment (g) and *W*_R_ is the weight of fabric afterwards (g).

### 2.3. Surface Analysis and Tensile Strength

The surface morphologies of the ultramicrofiber PET tricot fabrics before and after alkali treatment with 25% omf NaOH at 100 °C were analyzed by scanning electron microscopy (SEM, S-4300, Hitachi Co. Ltd., Tokyo, Japan). The physical properties of the alkali-treated samples were determined at a tensile rate of 100 mm/min using a universal testing machine (Instron 59802, Instron Co. Ltd., High Wycombe, UK), according to KS K 0521. The samples were cut into 50 × 300 mm^2^ pieces and the tensile strengths were calculated by dividing the maximum load by the cross-sectional area. Five samples were tested and the results averaged.

### 2.4. Dyeing

The dyeing characteristics of the PET ultramicrofibers treated with 25% omf NaOH aqueous solution at 100 °C for 30 min were investigated with a black disperse dye using the IR dyeing machine. Figure 1b shows the dyeing and reductive cleaning profiles for the sea-island-type PET ultramicrofiber tricot fabric. Dyeing was carried out for different times (20–60 min) and temperatures (95–135 °C). We prepared dyebaths comprising of the disperse dye (2–10 wt %), pH buffer solution (1–9 g/L), and UV-absorbent (5–25% omf). The goods-to-liquor ratio was 1:10. At the end of the dyeing process, to remove the unfixed dye, which would decrease the colorfastness, reductive cleaning was conducted by treatment with thiourea dioxide (2 g/L, CH_4_N_2_O_2_S, KC Chemicals(M) SDN BHD, Selangor, Malaysia) and sodium carbonate (4 g/L, Na_2_CO_3_, 99.5%, Aldrich Chemical Co., St. Louis, MO, USA) at 80 °C for 30 min. Dyed samples were rinsed five times under running water and oven-dried at 130 °C for 5 min.

### 2.5. Measurement of Color Yield and Colorfastness

The ratios of the absorption and scattering coefficients (*K*/*S*) of the dyed fabrics were measured using a ColorMate spectrophotometer (SCINCO Co., Ltd., Seoul, Korea) with D65 standard light illuminant and a 10° standard observer over the wavelength range of 400–750 nm. According to the Kubelka–Munk theory, the *K*/*S* value from the surface reflectance of the maximum absorption wavelength is given by Equation (2):

(2) $$K/S=\frac{{\left(1-R\right)}^{2}}{2R\;}$$

where *K* denotes the absorption coefficient, *S* is the scattering coefficient, and *R* indicates the reflectance (0 < *R* ≤ 1). Since the target color, black, is achromatic, it was evaluated by the lightness (L^*^) and color yield (*K*/*S*) values. Colorfastness to rubbing and light was measured using standard methods (KS K ISO 105-X12:2016 and KS K ISO 105-B02, respectively). The light colorfastness was evaluated according to the content of the UV-absorbent without any heat-treatment of the sample.

## 3. Results and Discussion

### 3.1. Alkaline Hydrolysis

**Figure 2:**
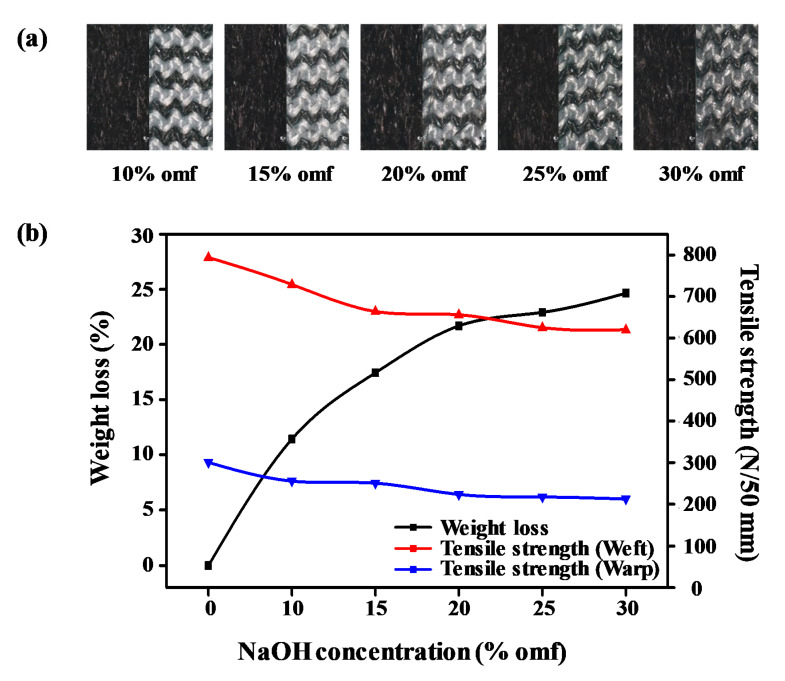
(**a**) Sea-island-type PET tricot fabrics treated at various NaOH concentrations (10–30% omf). (**b**) Weight loss ratios and tensile strengths of sea-island-type PET tricot fabrics treated at various NaOH concentrations (10–30% omf).

Alkaline hydrolysis is critical to reducing fabric weight as well as improving its texture and flexibility. The concentration of the NaOH aqueous solution exerts a significant effect on the weight loss ratio and physical properties [19,20]. In this study, the weight loss ratio (%) and tensile strength (N/mm) at each NaOH concentration were compared. Figure 2a shows photographic images of sea-island-type ultramicrofiber PET tricot samples treated at various NaOH concentrations. In Figure 2b, we observe that increasing NaOH concentration results in an increased weight loss ratio. Since the nucleophilic OH^−^ ions in NaOH dissociate the fibers, the weight loss ratio of the sea-island-type ultramicrofibers increases with rising NaOH concentration. In the reaction of alkaline hydrolysis, the highly nucleophilic OH^−^ ion randomly attacks the electron-deficient carbonyl group in the polyester and results in cleavage of the ester bond. After the ester bond breaking, hydroxyl groups and carboxyl groups are formed on the fiber surface, resulting in weight loss [24].

Since the mechanical properties such as tensile strength of the alkali-treated fabric must be maintained for the final product application, the tensile strength of the weight-reduced fabric was investigated to optimize the NaOH concentration. Table 1 and Figure 2b present the effects of the NaOH concentration on the sea-island ultramicrofiber PET tricot fabric at 100 °C for 30 min at a goods-to-liquor ratio of 1:10. The tensile strengths of the untreated fabric in the weft and warp directions were highest at 794 and 307 N/50 mm, respectively, compared with the alkali-treated fabrics. The tensile strengths of the alkali-treated fabrics declined gradually with increasing NaOH concentration. The ‘sea’ components were extracted with increasing NaOH concentration until 25% omf NaOH, and damage of the ‘island’ components above 25% omf NaOH led to a reduction in tensile strength. To determine the optimum conditions for alkaline hydrolysis of the sea-island ultramicrofiber PET tricot fabric, SEM analyses were performed on the untreated fabric as well as materials treated at various NaOH levels.

### 3.2. SEM Analysis

**Figure 3:**
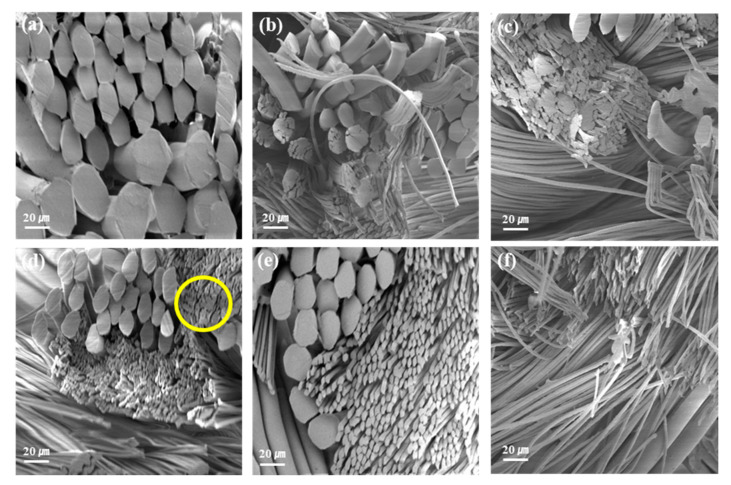
SEM images of sea-island ultramicrofibers of PET tricot fabrics before and after alkali treatment with various NaOH concentrations at 100 °C for 30 min: (**a**) untreated, (**b**) 10% omf NaOH, (**c**) 15% omf NaOH, (**d**) 20% omf NaOH, (**e**) 25% omf NaOH, and (**f**) 30% omf NaOH.

Figure 3 displays the SEM images of the sea-island ultramicrofibers of the PET tricot fabrics before and after alkali treatment at various NaOH concentrations (100 °C, 30 min). From the SEM images, most of the ‘island’ components were separated due to extraction of the ‘sea’ components at NaOH concentrations above 25% omf. At 20% omf (Figure 3d), several outer ‘island’ parts appeared separated in the cross-sectional view, which could be attributed to dissolution of the ‘sea’ components, but the interior parts show little separation. At 25% omf NaOH (Figure 3e), complete extraction of the ‘sea’ component was observed. At 30% omf NaOH (Figure 3f), the sea component was excessively dissolved. Thus, the hydrolysis condition of 25% omf NaOH with an average weight loss of 23.47% was selected as optimal.

### 3.3. Effect of Temperature

**Figure 4:**
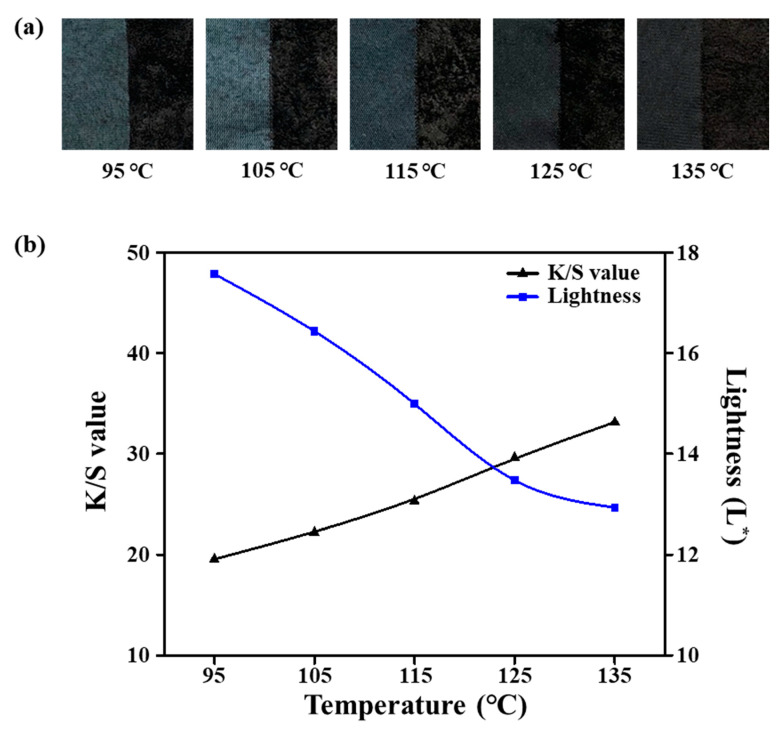
(**a**) Dyed samples. (**b**) *K*/*S* and lightness (L*) values according to the dyeing temperature (95–135 °C).

Generally, the dyeing of ultramicrofiber PET tricot fabrics with disperse dyes takes place through adsorption via hydrogen bonds and van der Waals forces. The disperse dyes penetrate and diffuse into amorphous regions of the polymers loosened by thermal motion at temperatures. Therefore, the dyeing temperature is an important factor affecting the dyeability. To investigate the effect of heating on dyeing characteristics, we set the dyeing temperature in the range of 95–135 °C. Figure 4a shows photographic images of sea-island-type PET tricot fabric samples dyed at various temperatures. These samples were alkali-treated under the optimal conditions at 100 °C in 25% omf NaOH for 30 min, and then dyed with 8% omf disperse dye, 1 g/L dispersant, 1 g/L pH buffer solution, and 10% omf UV-absorbent at a 1:10 liquor ratio for 40 min. When the dyeing temperature increased, the lightness value L* decreased from 17.57 to 12.93, whereas the *K*/*S* value increased from 19.55 to 33.14 (the *K*/*S* value was evaluated at 540 nm), as shown in Figure 4b. These results indicate that dye diffusion or migration from the surface into the core of the fiber was more facile at higher temperature (135 °C). Since there were limitations to increasing the dyeing temperature above 135 °C in dyeing machine, we selected 135 °C as the optimum dyeing temperature for the sea-island ultramicrofiber PET tricot fabric.

### 3.4. Effect of Dyeing Time

**Figure 5:**
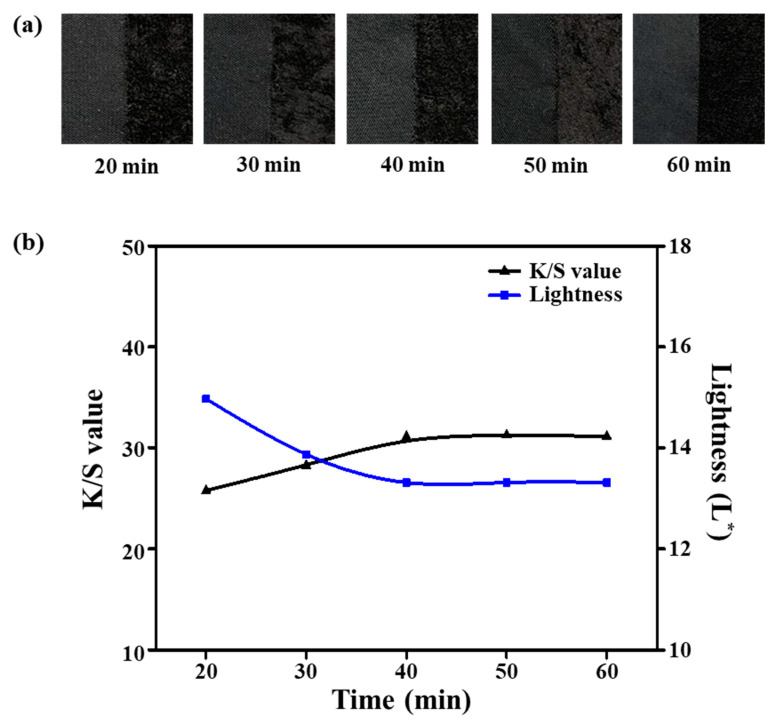
(**a**) Dyed samples. (**b**) *K*/*S* and lightness (L*) values according to dyeing time (20–60 min).

The process of dyeing fibers can be divided primarily into three steps: the dye moves into the surface of the fiber polymer in the dyebath solution; the dye is adsorbed on the fiber surface; and the adsorbed dye is diffused into the amorphous regions of the fiber. These dyeing steps are reversible and the isothermal adsorption behavior is generally linear [25]. To investigate the dyeing characteristics for different dyeing times (20–60 min) at 135 °C, the ultramicrofiber samples were first treated with 25% omf NaOH aqueous solution at 100 °C for 30 min and then dyed with 8% omf disperse dye, 1 g/L dispersant, 1 g/L pH buffer solution, and 10% omf UV-absorbent at a 1:10 liquor ratio. Figure 5a displays images of the dyed samples as a function of dyeing time, and Figure 5b shows the lightness and *K*/*S* values according to the various dyeing times. The *K*/*S* values increased from 25.75 to 31.13, and the lightness decreased from 14.23 to 12.19. Although both metrics changed rapidly initially, there were no significant changes in the L^*^ and *K*/*S* values after 40 min dyeing. When the ultramicrofibers of the PET tricot fabric were dyed for more than 40 min, we would expect that the desorption rate of the dye molecules would be similar to the adsorption rate on the fiber surface. These results indicate that the sea-island ultramicrofiber PET tricot fabric reached the equilibrium state at 40 min. Thus, the optimal dyeing time was 40 min.

### 3.5. Effect of Dye Concentration

**Figure 6:**
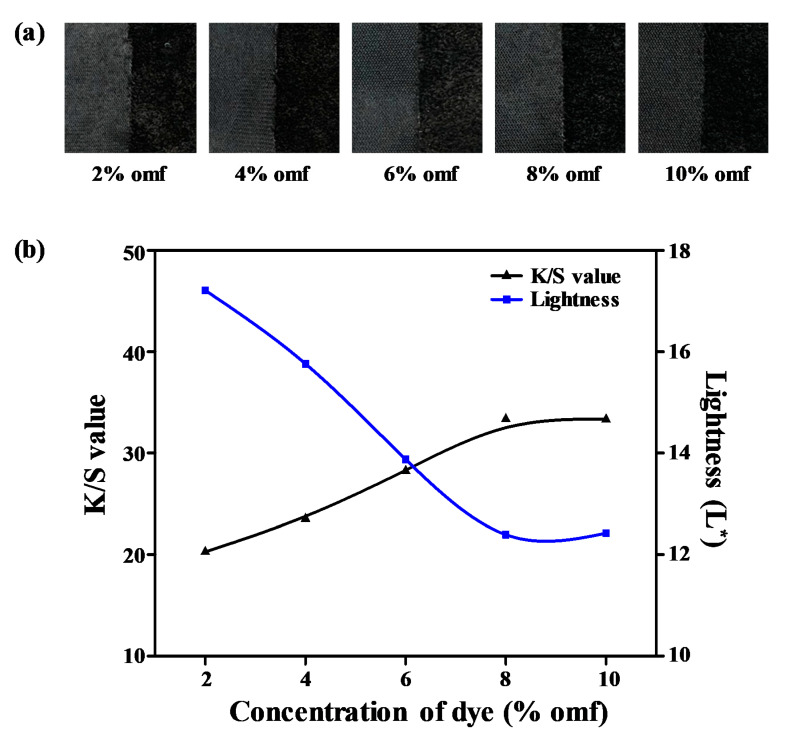
(**a**) Dyed samples. (**b**) *K*/*S* and lightness (L*) values according to dye concentration (2–10% omf).

If one compares ultramicrofiber PET tricot fabrics with general PET fibers, the former has a larger specific surface area than the latter. Hence, the ultramicrofiber PET tricot fabric needs a large amount of dye to produce a deep color. However, a large amount of the dye results in low colorfastness to light, washing, and rubbing. To further understand the effect of the black disperse dye concentration (2–10% omf), we investigated the dye’s build-up properties when the optimum dyeing temperature was fixed at 135 °C for 40 min with 1 g/L dispersant and 1 g/L pH buffer solution at a 1:10 liquor ratio. Figure 6a shows images of the dyed samples with various dye concentrations, and Figure 6b displays the lightness and *K*/*S* values according to the various dye concentrations. The L^*^ values decreased from 17.21 to 12.42, and the *K*/*S* values increased from 20.26 to 33.31 as the dye concentration increased. In the range of dye concentrations from 2% to 8% omf, excellent build-up properties were observed because the amount of dye adsorbed in the amorphous regions of the fiber increased. However, there were no significant changes in the dyeability above a dye concentration of 8% omf. Even if a large amount (10% omf) of disperse dye was dissolved in the dyebath, the absorption of dye into the fiber became limited. Hence, the optimal dye concentration was 8% omf for the alkali-treated sea-island ultramicrofiber PET tricot fabric.

### 3.6. Effect of Dispersant Concentration

**Figure 7:**
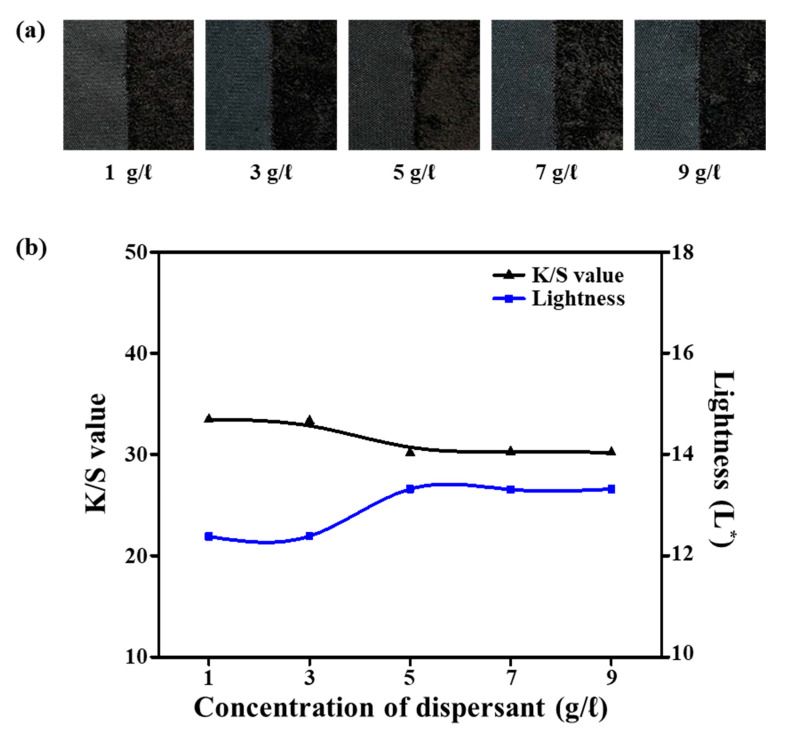
(**a**) Dyed samples. (**b**) *K*/*S* and lightness (L*) values according to dispersant concentration (1–9 g/L).

Generally, a large amount of water is essential to dye PET fibers, and a dispersant of 50–70% is used to solve the disproportionate problem caused by the initial high absorption rate of ultramicrofiber PET tricot fabric and to improve dispersibility. However, using a large amount of dispersants is environmentally undesirable [26]. To investigate the effect of dispersant concentration on dyeing properties, we employed concentrations of 1–9 g/L in the dyeing of the alkali-treated (25% omf NaOH at 100 °C for 30 min) sea-island ultramicrofiber PET tricot fabric. The other dyeing parameters were fixed at 135 °C for 40 min with 8% omf dye, 1 g/L pH buffer solution, and 10% omf UV-absorbent at a 1:10 liquor ratio. Figure 7a presents images of the dyed samples according to the various dispersant concentrations, and Figure 7b shows the L^*^ and *K*/*S* values according to the dispersant concentration. As the amount of dispersant increased, L^*^ increased from 12.38 to 13.31 while *K*/*S* decreased from 33.44 to 30.18, which did not significantly affect the black coloration of the sea-island ultramicrofiber PET tricot fabric. Since a large amount of fabric is required to evaluate the effect of dispersant concentration, the assessment of the effective amount of dispersant at the laboratory scale is inadequate. However, since dispersants are essential to prevent uneven dyeing on industrial scale, we selected a dispersant concentration of 1 g/L.

### 3.7. Effect of pH Buffer Solution Concentration

It is well known that the pH of the dyebath affects the *K*/*S* and lightness values as well as the absorption characteristics. The optimal pH of the dyebath is typically in the 4–6 range to obtain excellent dyeability on the sea-island ultramicrofibers of PET tricot. The dispersion stability of the disperse dye worsens when the pH of the dyebath is higher than 6 [27].

**Figure 8:**
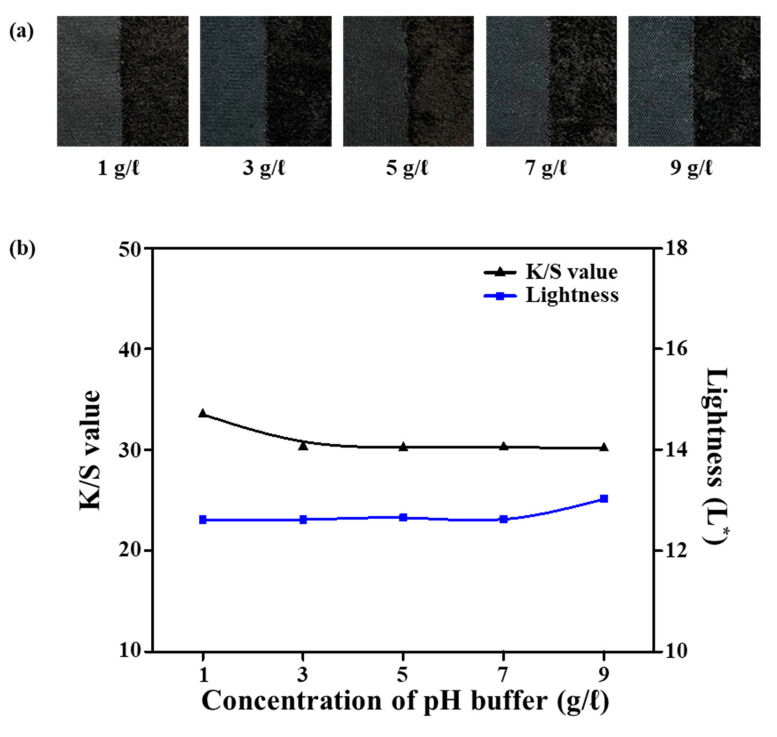
(**a**) Dyed samples. (**b**) *K*/*S* and lightness (L*) values according to pH buffer solution concentration (1–9 g/L).

To investigate the dyeing characteristics with pH buffer solution concentrations from 1 to 9 g/L, the sea-island ultramicrofiber PET tricot fabrics were first alkali-treated with 25% omf NaOH aqueous solution at 100 °C for 30 min and then dyed with 8% omf disperse dye, 1 g/L dispersant, and 10% omf UV-absorbent at a 1:10 liquor ratio at 135 °C for 40 min. Figure 8a shows images of the dyed samples according to the various pH buffer solution concentrations. Figure 8b shows the L^*^ and *K*/*S* values according to the pH buffer solution concentration. For buffer solution concentrations of 1, 3, 5, 7, and 9 g/L, the pH values of the dyebath are 5.30, 5.02, 4.90, 4.78, and 4.71, respectively. As the concentration of the pH buffer solution increased from 1 to 9 g/L, L^*^ increased from 12.61 to 13.02 and *K*/*S* decreased from 33.5 to 30.15. It is considered that the dispersion stability of the black disperse dye was influenced by the pH of the dyebath. As the concentration of the pH buffer solution was increased, the dispersion stability was reduced, resulting in poorer dyeability. These results suggest that the optimum concentration of the pH buffer solution was 1g/L.

### 3.8. UV-Absorbent Concentration Effect and Light Colorfastness Analysis

**Figure 9:**
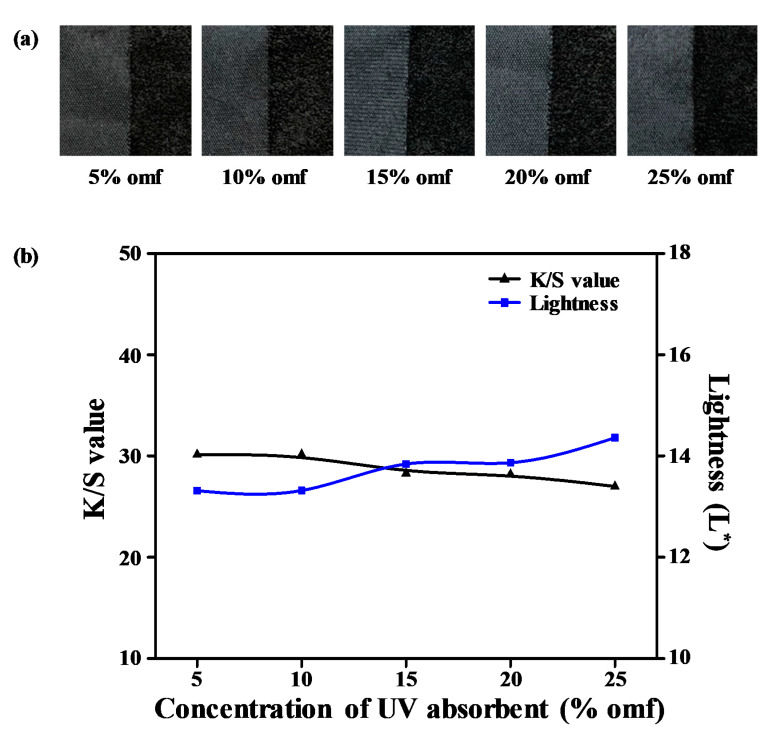
(**a**) Dyed samples. (**b**) *K*/*S* and lightness (L*) values according to UV absorbent concentration (5–25% omf).

To determine the optimal amount of UV-absorbent to obtain excellent light colorfastness on the sea-island ultramicrofiber PET tricot fabric, we conducted dyeing by adjusting the amount of the UV-absorbent to 5–25% omf after alkaline treatment with 25% omf NaOH aqueous solution at 100 °C for 30 min. Dyeing was performed with 8% omf disperse dye, 1 g/L dispersant, and 1 g/L pH buffer solution concentration at 1:10 goods-to-liquor ratio at 135 °C for 40 min. Figure 9a presents photographic images of the dyed samples according to the varying UV-absorbent contents. Figure 9b displays the L^*^ and *K*/*S* values according to the different amounts of UV-absorbent (5–25% omf). As the amount of UV-absorbent increased, the lightness value increased from 13.31 to 14.36, while that of *K*/*S* decreased from 30.13 to 26.98. The amount of dye that can be adsorbed on the fabric decreases inversely with the amount of UV-absorbent because of the reaction between the acidic phenol groups of the benzotriazole-type UV-absorbent and the amino groups of the disperse dye [28]. The optimum amount of UV-absorbent for dyeing was thus established at 10% omf.

As shown in Table 2, we also conducted a light colorfastness analysis according to the various contents of the UV-absorbent, which confirmed that all dyed samples had excellent light colorfastness of 4–5 grades. Overall, the optimal UV-absorbent content for producing excellent light colorfastness was 10% omf.

### 3.9. Rubbing Colorfastness

The rubbing colorfastness was evaluated for the alkali-treated ultramicrofiber PET tricot fabric dyed with disperse dye under the optimal conditions (8% omf dye, 1 g/L dispersant, 1 g/L pH buffer solution, and 10% omf UV-absorbent at 135 °C for 40 min at a 1:10 goods-to-liquor ratio). The excellent rubbing colorfastness results were related to the covalent bonds formed between the fibers and dye molecules. The rubbing colorfastness values in the dry and wet states spanned four grades and were indicative of superior colorfastness.

## 4. Conclusions

In this study, the alkaline hydrolysis and dyeing of sea-island ultramicrofibers of PET tricot fabrics were investigated to obtain the optimum conditions for weight reduction and dyeing. The optimum alkaline hydrolysis conditions were established at 25% omf NaOH at 100 °C for 30 min. To investigate the effects of this treatment on dyeability, we conducted the dyeing process under different conditions (i.e., varying the temperature, time, pH buffer solution concentration, and the amounts of disperse dye, dispersant, and UV-absorbent). The most appropriate dyeing conditions for the alkali-treated fabric in a 1:10 goods-to-liquor ratio were 8% omf disperse dye, 1 g/L dispersant, 1 g/L pH buffer solution, and 10% omf UV-absorbent at 135 °C for 40 min. The light and rubbing colorfastness values of samples dyed under the optimal conditions were excellent, with 4–5 grades for light colorfastness and four grades under wet and dry conditions for rubbing colorfastness.

## Figures and Tables

**Table 1:** Tensile strength retention of sea-island-type ultramicrofiber tricot fabrics treated with various NaOH concentrations at 100 °C.

Sample	Tensile Strength (N/50 mm)		Tensile Strength Retention (%)	
	Weft	Warp	Weft	Warp
No treatment	794	307	100	100
NaOH 10% omf	729	262	91.81	85.34
NaOH 15% omf	664	257	83.63	83.71
NaOH 20% omf	656	230	82.62	74.92
NaOH 25% omf	625	224	78.72	72.96
NaOH 30% omf	620	219	78.09	71.34

**Table 2:** Light colorfastness (KS K ISO 105-B02: 2014) of dyed fabrics according to the concentration of UV-absorbent (5–25% omf).

Concentration of UV-Absorbent (% omf)	Light Fastness (Grade)
5	4–5
10	4–5
15	4–5
20	4–5
25	4–5
